# Beliefs and Knowledge about Vaccination against AH1N1pdm09 Infection and Uptake Factors among Chinese Parents

**DOI:** 10.3390/ijerph110201989

**Published:** 2014-02-14

**Authors:** Cynthia Sau Ting Wu, Enid Wai Yung Kwong, Ho Ting Wong, Suet Hang Lo, Anthony Siu Wo Wong

**Affiliations:** 1 School of Nursing, the Hong Kong Polytechnic University, Hong Kong, SAR, China; E-Mails: enid.kwong@polyu.edu.hk (E.W.Y.K.); frank.ht.wong@polyu.edu.hk (H.T.W.); anthony.sw.wong@polyu.edu.hk (A.S.W.W.); 2 Princess Margaret Hospital, Hospital Authority, Hong Kong, SAR, China; E-Mail: lo.suethang@gmail.com

**Keywords:** Chinese parents, community care, health belief model, influenza vaccination, parents, preschool children

## Abstract

Vaccination against AH1N1pdm09 infection (human swine infection, HSI) is an effective measure of preventing pandemic infection, especially for high-risk groups like children between the ages of 6 months and 6 years. This study used a cross-sectional correlation design and aimed to identify predicting factors of parental acceptance of the HSI vaccine (HSIV) and uptake of the vaccination by their preschool-aged children in Hong Kong. A total of 250 parents were recruited from four randomly selected kindergartens. A self-administered questionnaire based on the health belief framework was used for data collection. The results showed that a number of factors significantly affected the tendency toward new vaccination uptake; these factors included parental age, HSI vaccination history of the children in their family, preferable price of the vaccine, perceived severity, perceived benefits, perceived barriers, and motivating factors for taking new vaccines. Using these factors, a logistic regression model with a high Nagelkerke R^2^ of 0.63 was generated to explain vaccination acceptance. A strong correlation between parental acceptance of new vaccinations and the motivating factors of vaccination uptake was found, which indicates the importance of involving parents in policy implementation for any new vaccination schemes. Overall, in order to fight against pandemics and enhance vaccination acceptance, it is essential for the government to understand the above factors determining parental acceptance of new vaccinations for their preschool-aged children.

## 1. Introduction

In recent years, there have been outbreaks of infectious diseases such as severe acute respiratory syndrome (SARS), avian influenza, and AH1N1pdm09 infection (human swine infection, HSI). HSI was classified as a statutorily notifiable disease in Hong Kong on April 27, 2009, after the World Health Organization (WHO) declared that the HSI outbreak had reached global pandemic status [1,2]. During the outbreak in Hong Kong, a high number of children under the age of 6 years required hospitalization; they accounted for 34.2% of overall hospitalizations associated with HSI by the end of 2009. In March 2010, the Hong Kong Centre for Health Protection (CHP) stated that 25% of patients who tested positive for HSI were preschool-aged children [3]. It is also important to note that about two-thirds of all patients confirmed with HSI were below 20 years of age [3], and that patients in this age group are at higher risk of developing serious complications when infected. 

Vaccination is a viable option to reduce the chance of hospitalization and death due to this infection [3,4]. Vaccination against H1N1pdm09 infection (HSIV) is an effective measure for preventing and combating an HSI pandemic, and for high-risk groups like children between the ages of 6 months and 6 years, it is offered free of charge by the government based on the recommendations of the Hong Kong Government’s Scientific Committees under the Center for Health Protection. For non-target groups, 500,000 doses of vaccine were made available at private clinics at a cost of HK$79 per dose (excluding injection fees) [4].

If the community is informed by the government and engaged in pandemic preparation, this could lead to a community response to government initiatives. A previous study mentioned that providing accurate pandemic-related information to the public is essential, and the public should work with the government in partnership in order to plan for pandemics [5]. During the SARS outbreak, for instance, poorly understood control measures caused confusion and fear [6], and similarly to SARS, HSI needs to be treated as an emergency. However, as HSI poses both a serious threat and has poorly understood effects, people are confused about and fearful of the new vaccination program. Based on the existing knowledge, a knowledge gap has been identified between parental acceptance and HSIV. However, research findings from overseas may not be applicable to the Hong Kong situation. Therefore, a local study was needed to investigate the factors influencing parental acceptance of HSIV in Hong Kong, which is one of the most important regions in China.

The aim of this study was to identify the predictors for parental acceptance of a new vaccination and uptake of the vaccination in preschool-aged children in Hong Kong based on the Health Belief Model (HBM) [7]. This model focuses on an individual’s threat perception of a health problem and the appraisal of recommended behavior(s) for preventing or managing the problem; the stages of the change model focus on the individual’s readiness to make a change or attempt to make a change toward healthy behaviors [8]. It enables a systematic and deep understanding of acceptance of immunization [9,10]. Basically, the model is grounded on the following four the concepts: (1) perceived susceptibility, which expresses one’s opinion of the chances of getting a disease; (2) perceived severity, which refers to one’s opinion of the seriousness of a condition and its long-term effects; (3) perceived benefits, or one’s opinion of the efficacy of the advised action to reduce the risk or seriousness of the impact of a disease by taking a vaccine; and (4) perceived barriers, which are one’s opinion of the tangible and psychological costs of the advised action in taking a vaccine [11]. 

## 2. Methods

### 2.1.Research Design and Study Sample

A cross-sectional correlation design was adopted to conduct the investigation. A total of 250 parents from four kindergartens, which were randomly selected from the available school list, were recruited. The school list, which included all kindergartens in Hong Kong, was obtained from the Education Bureau, so it is expected that the kindergartens covered families of all different socioeconomic statuses. Four kindergartens with not less than 100 children were selected from the four different areas of Hong Kong, which were Hong Kong East, Hong Kong South, Hong Kong West, and Hong Kong North. All of the kindergartens were located near public estates and parents that fulfilled the inclusion criteria of having children between the ages of 6 months and 6 years were selected. 

### 2.2. Instruments

The questionnaire was based on the health belief framework and a study conducted by Mok *et al.* [12]. The self-administered survey consisted of six parts: The first consisted of eight questions on demographic variables and one question on related chronic diseases in the children (Table 1), while the second included one question on the preferable price of HSIV (Table 2). The third contained 16 questions on health-belief measures and used a 5-point Likert scale; the health-belief measure included four questions on perceived susceptibility of HSI, three questions on perceived severity of HSI, four questions on perceived benefits of HSIV, and five questions on perceived barriers to taking HSIV (Table 3). The fourth part involved three questions that tested the respondents’ knowledge of HSIV (Table 4) and one question on the perceived knowledge about HSIV (Table 2). The fifth part included five questions on motivating factors for vaccination uptake (Table 4). The final part involved one question asking if the children had received a HSIV shot, and the reply was used to divide the subjects into groups that accepted HSIV and that were against HSIV. This was the primary outcome of this study.

Content validity was assured by conducting an expert review of the self-administered questionnaire. Three researchers in public health and community nursing, as well as advanced practice nurses in child and adolescent health were invited to assess the content validity index (CVI) [13]. A finalized questionnaire with a scale level CVI of 0.8 was obtained by averaging the item level CVI for all items in the scale. The result means that 80% of the total items were judged content valid, which is considered as acceptable [13]. The questionnaire was also pilot tested on 10 subjects for ensuring its validity and reliability. In the full-scale study, the Cronbach’s alpha was used for assessing the internal consistency of the HBM subscales. Theoretically the Cronbach’s alpha ranges from 0 to 1, and a higher value means better internal consistency of the scale. It is suggested that values within 0.8–0.9 is the most ideal [14]. For the HBM subscales in this study, their Cronbach’s alpha ranged from 0.65 to 0.84 (perceived susceptibility: 0.81; perceived severity: 0.79; perceived benefits: 0.84; perceived barriers: 0.65), while the Cronbach’s alpha of the scale concerning motivating factors of vaccination uptake was 0.93. 

### 2.3. Data Collection

A total of 250 questionnaires were distributed to parents of children studying in the selected kindergartens mentioned in section 2.1. Each eligible parent received one questionnaire, an information sheet, and a pre-addressed return envelope. Parents were told to return the completed questionnaires sealed in the envelopes provided before the designated deadline by submitting them to the class teacher. The researcher collected the completed questionnaires via class teachers at the kindergartens. The 118 questionnaires were collected at the end of July 2010 and the response rate was 47.2%.

### 2.4. Statistical Analysis

The Statistical Package for Social Sciences (SPSS) version 20 (SPSS Inc., Chicago, IL, USA) was used for data analysis. The impact of variables related to demographics, preferable price of HSIV, perceived and actual parental knowledge of HSIV, and parental acceptance of HSIV were examined using the Chi-square test/Fisher exact test. The differences in the variables of the HBM and motivating factors of vaccination taking between parents who accepted or rejected (did not accept) HSIV were compared using Mann-Whitney U test. Factors with p-values less than 0.2 obtained from the above bivariate analysis were further examined with a multivariate logistic regression analysis and forward stepwise (Wald) variable selection method to identify predictors of HSIV acceptance. The statistical significance was set to *p* = 0.05.

### 2.5. Ethical Considerations

This study was approved by the Human Subjects Ethics Committees of the Hong Kong Polytechnic University.

## 3. Results

### 3.1. Demographic Variables and HSIV

The subjects’ demographic information is shown in Table 1. Overall, 31.4% (n = 37) of the subjects accepted HSIV and 68.6% (n = 81) did not. Among the demographic variables, age of parents and HSI vaccination history of the children in their family were identified as significant differentiators between those accepting HSIV and those against it. The age of the parents in the HSIV group was significantly lower (Mode: 30–34 years) than in the group that did not accept it (Mode: 35–39 years; *p* < 0.05). A significantly larger proportion (98.8%) of the subjects in the group against HSIV had no HSI vaccination history of the children in their family, which stands in contrast to only 64.9% in the HSIV group (*p* < 0.001; Table 1). 

**Table 1:** Comparison of the sample characteristics between the groups accept and did not accept vaccination against AH1N1pdm09 Infection (HSIV).

Demographic Factors		Total	Accept HSIV	Against HSIV	*p*-Value	
		N = 118 (%)	N = 37 (%)	N = 81 (%)		
Parental relationship	Father	22(18.6)	8(21.6)	14(17.3)	0.575	
	Mother	96(81.4)	29(78.4)	67(82.7)		
Age of parent (years)	<30	25(21.2)	11(29.7)	14(17.3)	0.043	0.125
	30–34	35(29.7)	15(40.5)	20(24.7)		0.080
	35–39	37(31.4)	7(18.9)	30(37.0)		0.049
	≥40	21(17.8)	4(10.8)	17(21.0)		0.180
Age of children (years)	<3	13(11.0)	4(10.8)	9(11.1)	0.936 ^†^	1.000 ^†^
	3	27(22.9)	7(18.9)	20(24.7)		0.489
	4	33(28.0)	11(29.7)	22(27.2)		0.773
	>4	45(38.1)	15(40.5)	30(37.0)		0.716
HSI vaccination history of the children in their family	Yes	14(11.9)	13(35.1)	1(1.2)	0.000 ^†^	
	No	104(88.1)	24(64.9)	80(98.8)		
Education level	Primary or below	6(5.1)	1(2.7)	5(6.2)	0.210 ^†^	0.664 †
	Secondary	79(66.9)	29(78.4)	50(61.7)		0.074
	Tertiary or above	33(28.0)	7(18.9)	26(32.1)		0.139
Occupation	Health care professional	33(28.0)	14(37.8)	19(23.5)	0.106	
	Non-health care professional	85(72.0)	23(62.2)	62(76.5)		
^Monthly family income (HK$)	<5,000	32(27.6)	9(24.3)	23(29.1)	0.277	0.591
	5,001–10,000	24(20.7)	11(29.7)	13(16.5)		0.100
	10,001–30,000	28(24.1)	6(16.2)	22(27.8)		0.172
	>30,000	32(27.6)	11(29.7)	21(26.6)		0.724
Religion	Catholic	4(3.4)	0(0.0)	4(4.9)	0.555 ^†^	0.307 ^†^
	Christian	12(10.2)	5(13.5)	7(8.6)		0.513 ^†^
	Buddhist	6(5.1)	2(5.4)	4(4.9)		1.000 ^†^
	No	96(81.4)	30(81.1)	66(81.5)		0.959
Chronic disease	Yes	6(5.1)	2(5.4)	4(4.9)	1.000 ^†^	
	No	112(94.9)	35(94.6)	77(95.1)		

Note*:* ^n = 79 for the against HSIV group; ^†^ Fisher exact test.

### 3.2. Preferred Price of HSIV and Perceived Knowledge of HSIV

Preferable price of HSIV, perceived knowledge of HSIV, and the corresponding HSIV acceptance rates are shown in Table 2. More than half of the subjects preferred HSIV to be free, and 66.1% (n = 78) perceived that knowledge of HSIV was insufficient at best. There were no significant differences in perceived knowledge of HSIV between the two groups. However, there was a significantly higher proportion of parents in the group against HSIV that preferred the vaccine to be provided free of charge (*p* < 0.05). This eliminated the possibility that parents might be against the vaccine due to the concern of vaccine quality if it is free of charge.

**Table 2:** Comparison of the preferable price and perceived knowledge of vaccination against AH1N1pdm09 Infection (HSIV) between the groups accept and did not accept HSIV.

		Total	Accept HSIV	Against HSIV	*p*-Value	
		N = 118 (%)	N = 37 (%)	N = 81 (%)		
Preferable price receiving HSIV (HK$)	Free of charge	66 (55.9)	15 (40.5)	51 (63.0)	0.016 ^†^	0.023
	<50	30 (25.4)	14(37.8)	16(19.8)		0.036
	50–100	16 (13.6)	6(16.2)	10(12.3)		0.569
	101–150	4(3.2)	0(0.0)	4(4.9)		0.307 ^†^
	150+	2(1.7)	2(5.4)	0(0.0)		0.096 ^†^
Perceived knowledge of HSIV	Very insufficient	9(7.6)	2(5.4)	7(8.6)	0.182 ^†^	0.718 ^†^
	Insufficient	69(58.5)	18(48.6)	51(63.0)		0.143
	Sufficient	38(32.2)	17(45.9)	21(25.9)		0.031
	Very sufficient	2(1.7)	0(0.0)	2(2.5)		1.000 ^†^

Note: ^†^ Fisher exact test.

### 3.3. Health Belief Variables in the Two Groups

The results of the Mann-Whitney U test of the health belief variable between the two groups are shown in Table 3. Significant differences between the groups were observed in all domains except perceived susceptibility to HSI. Particularly for perceived severity of HSI, it was found that parents whose children had received HSIV had a stronger belief that HSI could worsen underlying medical conditions in children (*p* < 0.05). 

**Table 3:** Comparison of the belief of human swine influenza between the groups accept and did not accept vaccination against AH1N1pdm09 Infection (HSIV).

	Accept HSIV	Against HSIV		*p*-Value ^†^	
	(n = 37)	(n = 81)			
	Mean	SD	Mean	SD	
**1. Perceived susceptibility to HSI**					
1.1 Compared with other children your child’s age, your child is more likely to get the flu	2.35	1.09	2.11	0.96	0.308
1.2 Children who do not get HSIV could get a more severe case of Human Swine Influenza	2.97	1.19	2.56	1.07	0.089
1.3 Kindergarten children are more likely to get HSI than adults	3.7	1.18	3.28	1.1	0.050
1.4 My child is likely to get the HSI.	2.68	1.16	2.41	1.01	0.275
**2. Perceived severity of HSI**					
2.1 HSI is more serious in a healthy child than in a healthy adult	3.43	1.17	3.22	1	0.348
2.2 If my children were to catch the Human Swine Influenza, it would be significantly interfere with his or her daily activities	4.05	0.97	3.9	1	0.423
2.3 The Human Swine Influenza can worsen the underlying medical conditions in children	4.05	1	3.62	1.02	0.023
**3. Perceived benefits of HSIV**					
3.1 HSIV can prevent my children from catching HSI	3.7	0.74	2.96	1.04	0.000
3.2 HSIV can prevent my children from catching Influenza	3.05	1.1	2.28	0.93	0.000
3.3 The immune system against the flu will be strengthened	3.43	0.93	2.46	1	0.000
3.4 Having HSIV can reduce the school absence rate of children	3.46	1.04	2.48	1.09	0.000
**4. Perceived barriers of HSIV**					
4.1 The HSIV injection is unsafe for the children	3.14	0.67	3.49	0.91	0.032
4.2 The information of HSIV is not clear enough	3.54	0.69	3.81	1.03	0.044
4.3 The HSIV will cause unknown side effects in children	3.22	0.85	3.79	0.89	0.001
4.4 I do not have time to bring my children to receive HSIV	2.19	1	2.35	1.23	0.661
4.5 The HSIV may cause HSI after injection	3.27	0.87	3.38	1.02	0.452

Note: ^†^ Mann-Whitney U test.

Similarly, there was a significantly higher degree of belief (*p* < 0.001) in all four statements regarding perceived benefits of HSIV between the participants accepting HSIV and those not accepting HSIV. Finally, for the domain of perceived barriers to taking HSIV, parents who were against HSIV had significantly stronger feelings than parents who accepted HSIV that the injection was unsafe for the child (*p* < 0.05), information on HSIV was not clear enough (*p* < 0.05), and HSIV could cause unknown side effects in children (*p* < 0.01).

### 3.4. HSIV Knowledge and Acceptance of HSIV

In the questionnaire section on HSIV knowledge, the statements “HSI causes tiredness, cough, sore throat, loss of appetite, diarrhea and fever,” and “Redness, soreness and swelling at the injection site are the common side effects of HSIV” were correct, whereas “People with egg allergies can get HSIV” was incorrect. Among the respondents, there was no significant relationship between the correctness of the assessment of above statements and the acceptance of HSIV (see Table 4).

**Table 4:** Comparison of knowledge and motivating factors of vaccination uptake between the groups accept and did not accept vaccination against AH1N1pdm09 Infection (HSIV).

`		Accept HSIV (N = 37)	Against HSIV (N = 81)	*p*-Value
**HSIV knowledge **		n (%)	n (%)	
1. HSI cause tiredness, cough, sore throat, loss of appetite, diarrhea and fever	Correctly answered	8 (21.6)	20 (24.7)	0.716
	Incorrectly answered	29 (78.4)	61 (75.3)	
2. People with egg allergies can get HSIV	Correctly answered	11 (29.7)	18 (22.2)	0.38
	Incorrectly answered	26 (70.3)	63 (77.8)	
3. Redness, soreness and swelling at the injection site are the common side effects of HSIV	Correctly answered	11 (29.7)	27 (33.3)	0.698
	Incorrectly answered	26 (70.3)	54 (66.7)	
**Motivating factors of vaccination uptake**		**Mean (SD)**	**Mean (SD)**	***p*-Value** ^†^
1. You would allow your children to receive HSIV if it was recommended by healthcare professional.		3.76 (0.8)	2.83 (1)	0.000
2. You would allow your children to receive HSIV if it was promoted by the government advertisement.		3.35 (0.79)	2.53 (0.95)	0.000
3. You would allow your children to receive HSIV if most of the parents you know took their children for HSIV shot.		3.65 (0.75)	2.93 (1.06)	0.000
4. You would be more willing to give your children HSIV shots if the injection was cheap.		3.62 (0.92)	2.51 (0.99)	0.000
5. You would be more willing to give your children HSIV if the vaccination location was easy to access.		3.57 (0.96)	2.62 (1.04)	0.000

Note: ^†^ Mann-Whitney U test.

### 3.5. Motivating Factors for Vaccination Uptake

Parents who accepted HSIV had a significantly stronger agreement (*p* < 0.001) in all five aspects of the motivating factors of vaccination taking than parents that belonged to the non-accepting group. The results are shown in Table 4. 

### 3.6. Predictors of Vaccination Acceptance

The logistic regression analysis showed that two aspects of the HBM (*i.e.*, perceived benefits and perceived barriers) and a motivating factor of vaccination taking were predictors for vaccine acceptance. 

To be more precise, the identified predictors were “HSIV can prevent my children from catching Influenza,” “HSIV will cause unknown side effects in children,” and “You would be more willing to give your children a HSIV shot if the injection was cheap.” The odds ratios were 2.367 (95% confidence interval [CI]: [1.272, 4.404]; *p* < 0.01), 0.321 (95%CI: [0.149, 0.691]; *p* < 0.01), and 3.675 (95%CI: [1.745, 7.742]; *p* < 0.01), respectively. The regression model also had a very high Nagelkerke R^2^ of 0.631(see Table 5).

**Table 5:** Predictors of vaccination against AH1N1pdm09 Infection (HSIV) acceptance.

Predictors	OR	95% CI		*p*-Value	Nagelkerke R^2^
Perceived benefits of HSIV:					0.631
HSIV can prevent children from catching Influenza	2.367	(1.272, 4.404)		0.007	
Perceived barriers of HSIV:					
The HSIV will cause unknown side effects to children	0.321	(0.149, 0.691)		0.004	
Motivating factors of vaccination taking:	3.675	(1.745, 7.742)		0.001	
You would be more willing to give your children HSIV shots if the injection was cheap					

Note: Sensitivity = 73%; Specificity = 91.4%; Overall = 85.6%; CI: Confidence Interval; OR: Odds Ratio; The following items were considered but finally removed by the Forward Stepwise (Wald) variable selection method from the regression model: Occupation; Preferable price receiving HSIV; Perceived knowledge of HSIV; Items 1.2, 1.3, 2.3, 3.1, 3.3, 3.4, 4.1, and 4.2 in Table 3; The first three and fifth motivating factors of vaccination uptake in Table 4; Significant demographic factors found in Table 1 were controlled in the model.

## 4. Discussion

### 4.1. Socio-Demographic Factors and Parental Acceptance of HSIV

In this study, the overall vaccination acceptance rate was 31.4% (n = 37), and this did not differ by education level. Some studies have also noted that education is not associated with the acceptance of vaccination in developed countries [15]. Although lower economic status was found to be associated with higher opposition to vaccinations in a prior study [16], there were no differences associated with economic status (monthly salary) in our study. A previous study found that parents in religious groups were more likely to have negative attitudes towards vaccination [17], but the findings of the present study showed that religious status did not influence HSIV acceptance.

On the other hand, this study did find two significant socio-demographic factors to be associated with parental acceptance of HSIV. Both of these were controlled in the logistic regression model discussed in section 3.6 to ensure the validity of the predictors found. Parents less than 35 years old were more likely to accept HSIV; the reasons for this are not clear, but it indicates that focusing educational efforts on older parents in particular—that is, those older than 34—may be beneficial. In addition, this study showed that having HSI vaccination history of the children in their family had a positive impact on parental acceptance of HSIV. The cause of this could be that parents realized the vaccine had a positive impact on their children, and thus became more likely to vaccinate their second and third children. This finding could help the government to increase vaccination rates, for example, by presenting vaccination as a social norm to encourage parents to vaccinate their first child. Social norms act as powerful influences in vaccination decision making; norms regarding what the parents’ social group considers to be “appropriate” health-related behavior have been identified as important factors in parental decision making about vaccines [18]. 

### 4.2. Preferable Price and HSIV

It is not surprising that there were significantly more parents that would consider accepting HSIV if it had lower cost. This was consistent with the results of a previous study, which predicted that the uptake of the vaccination against AH1N1pdm09 infection by the general population of Hong Kong was unlikely to be high and would be sensitive to personal cost [19]. When the vaccination scheme against AH1N1 pdm09 infection was announced by the Hong Kong government, children were provided free vaccinations in public clinics, and vaccinations performed in private clinics were subsidized. In private clinics, the government subsidized HK$129 per dose of the vaccine per child [4]. This indicates that the high-risk group of children was not fully protected by the limited subsidy coverage in private clinics. As some parents may be reluctant to seek services from a public clinic, all preschool children should receive full subsidization for the vaccination in order to achieve higher parental acceptance rates of HSIV. However, the factor of preferable price was ultimately excluded from the final logistic regression model. This is likely because the effect had already been reflected by one of the cost-related motivating factors of vaccination uptake retained in the regression model.

### 4.3. The Health Belief Model and HSIV

#### 4.3.1. Perceived Severity of HSI

In this study, a significant number of parents that accepted HSIV believed that HSI could worsen underlying medical conditions in their children. This could indicate that they believe HSI causes serious long-term effects in children. However, this health belief factor of perceived severity of HSI might be weak in determining parental acceptance of HSIV, as it is the only statements in the perceived severity of HSI section that was significant. The fact that it disappeared from the final logistic regression model also supports the above argument.

#### 4.3.2. Perceived Benefits of HSIV

All the statements under the domain of perceived benefits exhibited significant differences. Particularly, parents who accepted HSIV had overall mean scores ranging from 3.05 to 3.70, whereas parents who did not accept it only had mean scores ranging from 2.28 to 2.96. Such significant differences with a large effect size might indicate that this domain was relatively more important to the acceptance of HSIV. This is consistent with the regression model in Table 5, which also shows that this domain was one of the significant predictors. 

#### 4.3.3. Perceived Barriers to Taking HSIV

According to a number of studies, a lack of belief in the safety and efficacy of vaccines is the most commonly perceived barrier to vaccination [20,21]. Research on vaccination against the human papillomavirus among Californian parents showed similar results, in that perceived severity of the adverse effects following immunization and the perceived susceptibility to the disease were the most apparent parental decision factors for accepting the vaccine [22]. In this study, parents generally believed that HSIV was unsafe and that it would cause unknown adverse side effects in their children. Although only three of the five statements regarding the perceived barriers to taking HSIV were significant, the difference in the statement “HSIV will cause unknown side effects in children” was large. This result is consistent with previous studies, which also noted that the perceived barrier was not associated with immunization status, but instead related to the side effects of vaccines [23]. Furthermore, the statement also survived as a predictor in the final logistic regression model in Table 5.

Previous studies have indicated that the perceived barriers to vaccination included the efficacy of vaccinations, adverse effects of vaccinations, previous experiences with side effects of vaccinations, and a lack of belief in the safety of vaccinations [21,23]. In this study, the parents pointed out that the reasons for not accepting HSIV were that they questioned the efficacy of HSIV and were unsure about adverse effects. This strengthens the notion that the both of these factors are key elements in the perceived barriers to vaccination. It is important to note that the high perceived barriers to immunization were negative predictors [9]. Therefore, evidence on safety and efficacy is critical in increasing the uptake of vaccination [19]. 

### 4.4. Motivating Factors for Vaccination Taking

In this study, there was a significant difference among the groups (*p* < 0.001) regarding the statement “You would allow your children to receive HSIV if it was recommended by healthcare professional.” In a study on influenza vaccination, it was found that a doctor’s recommendation increased the likelihood of a patient accepting vaccination [24]. Therefore, HSIV should also be promoted by healthcare professionals to increase acceptance. 

Parents that accepted HSIV were significantly more likely to allow their children to receive HSIV if it was promoted by government advertisements (*p* < 0.001). This is consistent with other studies indicating that the media can have a significant influence on the attitudes and behaviors of the general public regarding a variety of health issues like lower back pain [25] or colon cancer screening [26].

Similarly, parents who accepted HSIV were significantly more likely to support children receiving HSIV if most of the parents they know take their children for an HSIV shot (*p* < 0.001). It was concluded that people have their children vaccinated because everybody does so and it seems the normal thing to do [18]. In previous studies, positive factors related to vaccination have included an increase in the perception that vaccination is the social norm [9]. 

Parents who accepted HSIV had a significantly stronger agreement (*p* < 0.001) with the statement “You would be more willing to give your children HSIV shots if the injection was cheap” than parents that did not accept HSIV. This result confirmed the findings of a previous study stating that the uptake of vaccination against AH1N1pdm09 infection by the general population of Hong Kong was unlikely to be high due to related personal cost [19]. Cost is thus confirmed as another important factor in vaccination uptake. 

Finally, there was a significant difference (*p* < 0.001) in responses to the statement “You would be more willing to give your children HSIV if the vaccination location was easy to access,” which is consistent with previous findings highlighting that easy access to the vaccine is crucial in increasing the vaccination rate [24]. 

Only the fourth statement, “You would be more willing to give your children HSIV shots if the injection was cheap,” was retained in the final regression model. The first three statements may have been excluded because they were related to the perceived severity of HSI and benefits of HSIV. The final accessibility-related statement, “You would be more willing to give your children HSIV if the vaccination location was easy to access,” was also excluded, possibly because travelling was also considered as a cost, which was represented by the fourth statement.

### 4.5. Predictors of Vaccine Acceptance

The obtained logistic regression model was promising due to the Nagelkerke R^2^ of 0.63, which is considered a high value in the field of social sciences [27]. The three predictors found in this study belong to the categories of perceived benefits, perceived barriers, and motivating factors. These predictors will allow policymakers to formulate specific promotion programs to augment the vaccine acceptance rate. For example, based on the two significant predictors related to perceived benefits and barriers, the promotion program should emphasize evidence on the effectiveness of HSIV and help the public to understand that the probability of experiencing unknown adverse side effects is actually very low. Based on the predictor related to the motivating factors of vaccination uptake, the government could explore the feasibility of increasing the subsidies for HSI vaccinations.

## 5. Limitations

The small sample size could be a major limitation of this study. Peng *et al.* [28] mentioned that there is no clear, specific rule recommended for logistic regression. Hence, the sample size calculation was not conducted. However, some researchers have suggested that 10 observations are required for each predictor, with a minimum total sample size of 100 [28]. The sample size in this study just reached the minimum requirement of sample size 100, as there were five predictors in the logistic regression model. If the sample size is insufficient, the power of the regression model will decrease and certain predictors might be missed. However, the predictors found in this study were still valid. Nevertheless, this study provided data to understand the predictors of parental acceptance of HSIV. Future research could be improved by conducting a larger study through an increase in sample size.

## 6. Conclusions

Parents are the protectors of their children’s health, and thus serve as the primary gatekeepers for vaccinations [18]. It has been reported that parental attitudes and beliefs are the most powerful predictors of vaccination uptake for their children [29], so their acceptance of a vaccine significantly affects the vaccination rate of this high-risk group. This study found that parents’ age and their children’s previous experience with vaccination were significantly associated with parental acceptance of subsequent vaccinations. The price of vaccination was another factor that determined future vaccination uptake. The perceived severity of new vaccines, perceived benefits, and perceived barriers were also associated with parental acceptance of a new vaccine. The safety and efficacy of the vaccination were critical in determining the likelihood of taking the vaccine, as seen in the regression model. However, there was no significant relationship between parental knowledge about the new vaccine and its acceptance. The strong correlation between parental acceptance of a new vaccination and the motivating factors of vaccination taking indicated the importance of involving parents in the policy implementation of any new vaccination scheme. Overall, to fight pandemics and enhance vaccination acceptance, it is essential that the government understands the above factors determining parental acceptance of new vaccinations for their preschool-aged children.
